# Identification of PLK1-PBD Inhibitors from the Library of Marine Natural Products: 3D QSAR Pharmacophore, ADMET, Scaffold Hopping, Molecular Docking, and Molecular Dynamics Study

**DOI:** 10.3390/md22020083

**Published:** 2024-02-10

**Authors:** Nan Zhou, Chuangze Zheng, Huiting Tan, Lianxiang Luo

**Affiliations:** 1 The First Clinical College, Guangdong Medical University, Zhanjiang 524023, China; zaonan@gdmu.edu.cn (N.Z.); zcz7720031115@gdmu.edu.cn (C.Z.); tanhui@gdmu.edu.cn (H.T.); 2 The Marine Biomedical Research Institute, School of Ocean and Tropical Medicine, Guangdong Medical University, Zhanjiang 524023, China; 3 The Marine Biomedical Research Institute of Guangdong Zhanjiang, Zhanjiang 524023, China

**Keywords:** marine natural compounds, PLK1-PBD, 3D QSAR pharmacophore, molecular docking, scaffold hopping, virtual screening, molecular dynamics

## Abstract

PLK1 is found to be highly expressed in various types of cancers, but the development of inhibitors for it has been slow. Most inhibitors are still in clinical stages, and many lack the necessary selectivity and anti-tumor effects. This study aimed to create new inhibitors for the PLK1-PBD by focusing on the PBD binding domain, which has the potential for greater selectivity. A 3D QSAR model was developed using a dataset of 112 compounds to evaluate 500 molecules. ADMET prediction was then used to select three molecules with strong drug-like characteristics. Scaffold hopping was employed to reconstruct 98 new compounds with improved drug-like properties and increased activity. Molecular docking was used to compare the efficient compound abbapolin, confirming the high-activity status of [(14S)-14-hydroxy-14-(pyridin-2-yl)tetradecyl]ammonium,[(14S)-15-(2-furyl)-14-hydroxypentadecyl]ammonium and [(14S)-14-hydroxy-14-phenyltetradecyl]ammonium. Molecular dynamics simulations and MMPBSA were conducted to evaluate the stability of the compounds in the presence of proteins. An in-depth analysis of [(14S)-15-(2-furyl)-14-hydroxypentadecyl]ammonium and [(14S)-14-hydroxy-14-phenyltetradecyl]ammonium identified them as potential candidates for PLK1 inhibitors.

## 1. Introduction

The polo-like kinase (PLK) family is a group of serine/threonine protein kinases that play a crucial role in regulating the cell cycle in eukaryotes. This family consists of five members: PLK1–PLK5, with PLK1 being the most extensively studied for its regulatory functions and potential as a drug target. PLK1 has a structure similar to other kinases, with a serine/threonine kinase domain at the N-terminal and a polo-box domain (PBD)-repeat sequence at the C-terminal. It contains two drug-binding sites that can be targeted for inhibitor design. PLK1 is essential for regulating mitosis, controlling cell entry into mitosis, phosphorylating and regulating key proteins, and performing other important tasks during the G2/M phase. Increased expression of PLK1 is observed in malignant cells, leading to defects in mitosis and cytokinesis as well as increased chromosomal instability, which is a common feature of many cancers [1]. A recent study found that inhibiting PLK1 kinase activity reduces the binding of UHRF1 and USP7, leading to the rapid breakdown of UHRF1 via the ubiquitin-proteasome pathway. This results in reduced recruitment of DNMT1 to chromatin, leading to decreased DNA methylation and increased expression of the tumor-suppressor gene TSG, ultimately slowing cell proliferation and promoting cell senescence [2]. In another study, it was discovered that PLK1 is essential for controlling tumor autophagy, and blocking PLK1 activity can restrict tumor growth by regulating autophagy [3]. PLK1 is typically expressed at low levels in healthy tissues but is found to be highly expressed in various types of cancer, such as breast and gastric cancer. Its high expression has been linked to a poor prognosis, making it a potential target for the development of cancer drugs. Several drugs have been developed to target PLK1, including BI2536, which targets the ATP-binding site (kinase domain) [4]. However, clinical trials have shown that its anti-tumor effect is low, leading to the discontinuation of its development. Volasertib is currently in phase 3 clinical trials and has shown the most progress, but most inhibitors targeting this binding domain are still in the preclinical stage and have had limited success. This may be due to a lack of specificity and the resulting dose-limiting toxicity [5]. By combining PBD domain drugs, it is feasible to attain enhanced selectivity at non-toxic doses by targeting various binding sites, thereby decreasing off-target effects. Additionally, allosteric inhibitors can address issues arising from mutations in the conserved ATP binding site by inhibiting proteins in an inactive state. Moreover, the potential for selectivity is greater when targeting protein–protein interactions with PBD [6,7]. This experiment was designed to identify inhibitors that specifically target the PBD-binding domain and that have a good affinity and selectivity.

Combined annotation-dependent depletion (CADD) utilizes computational methods to discover, design, and analyze drugs and molecules that share similar biochemical properties. This method has been instrumental in creating more than 70 commercial drugs [8]. CADD has been proven to be a reliable and highly respected computational method that competes with experimental high-throughput screening in identifying and optimizing hits, with virtual screening being the primary technology behind it. Among these, quantitative structure–activity relationship (QSAR) analysis stands out as the most effective due to its high capacity, speed, and accuracy [9]. Molecular docking is a widely used method for determining how ligands bind to their receptors. It uses force field calculations, which are informed by quantum mechanics and experimental data, to estimate the binding energy. For more precise data on binding energy, ab initio methods like density functional theory (DFT) and molecular dynamics simulations can be utilized. Molecular dynamics simulations are especially useful, as they can produce multiple conformational snapshots to validate the docking results [10]. The integration of the aforementioned technologies has led to numerous successful studies, resulting in a higher rate of drug design hits, increased success rates, reduced R&D costs, and shorter R&D cycles. The root mean square deviation (RMSD) reflects the extent to which atoms deviate from their average positions, indicating the magnitude of motion for each atom. Furthermore, this article also employs scaffold hopping to identify superior candidate compounds and circumvent intellectual property protection.

Organisms that inhabit aquatic and terrestrial environments, including microorganisms, fungi, plants, and animals, are responsible for the production of natural products (NPs) or secondary metabolites. These have been utilized for medicinal purposes since ancient times and continue to play a crucial role in modern pharmacology. Marine habitats, in particular, are abundant sources of bioactive metabolites with toxic or deterrent properties, and the diverse nature of the environment promotes the production of complex and diverse chemicals that hold great potential for development into drugs that cannot be replicated by synthetic small molecules [11].

Marine natural products have a unique three-dimensional structure enabling them to accurately bind to active sites and exhibit distinctive biological activities. Their structural characteristics include an abundance of sp3-hybridized carbon, chiral centers, and condensed fatty rings as well as a higher proportion of carbon, hydrogen, oxygen, and nitrogen atoms compared to synthetic drugs [12]. Sponges, algae, and corals that are found in the marine environment have been identified as sources of novel secondary metabolites with distinct chemical structures. These novel secondary metabolites are essential for the production of anti-cancer drugs [13]. There are currently over 10 medications approved for treating various types of cancer, all of which are derived from molecules found in the marine environment. These drugs include cytarabine, nelarabine (the prodrug of ara-G), fludarabine phosphate (the prodrug of ara-A), plitidepsin, midostaurin, eribulin mesylate, brentuximab vedotin, polatuzumab vedotin, enfortumab vedotin, belantamab mafodotin, trabectedin, and lurbinectedin [11]. Taking advantage of marine compound libraries for drug discovery is likely to lead to the identification of novel and powerful drugs [14].

**Figure 1:**
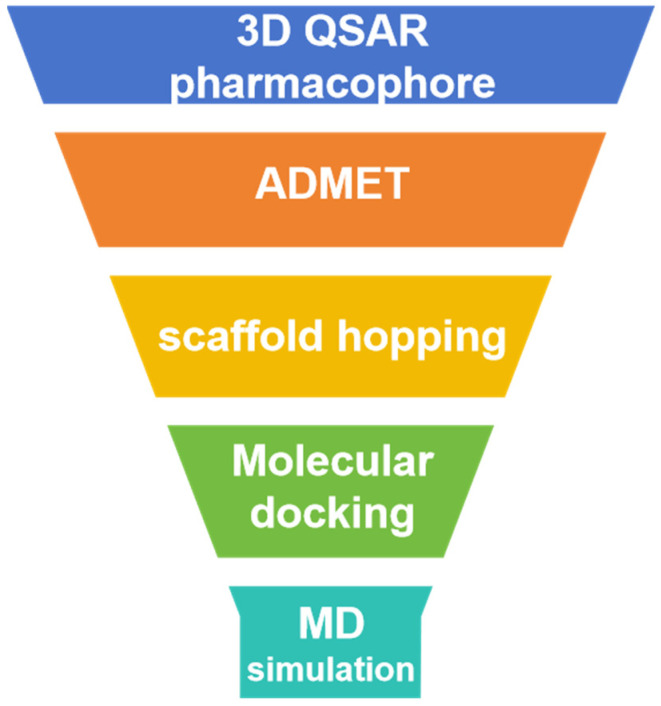
Virtual screening process for USP7 covalent inhibitors.

We obtained the *Marine Natural Products Database* (MNPD) [15] and employed a series of computer-aided methods to identify three small molecules from marine sources that have the potential to inhibit PLK1. Initially, we identified inhibitors for the PLK1-PBD domain and utilized 3D-QSAR pharmacophore modeling to evaluate and select the most promising pharmacophores. These pharmacophores were then used to predict activity and screen a marine library. The selected compounds were further evaluated for their absorption, distribution, metabolism, excretion, and toxicity as well as subjected to molecular docking to identify three molecules with improved activity and drug-likeness. To enhance their effectiveness, we performed scaffold hopping to modify the compounds and compared the before and after structural optimization using molecular docking. Finally, we conducted three molecular dynamics simulations on the optimized small molecules and identified effective PLK1 inhibitors based on the stability of the complexes. Figure 1 illustrates the workflow of this study.

## 2. Results

### 2.1. Construction, Selection, and Validation of Pharmacophore Model

**Figure 2:**
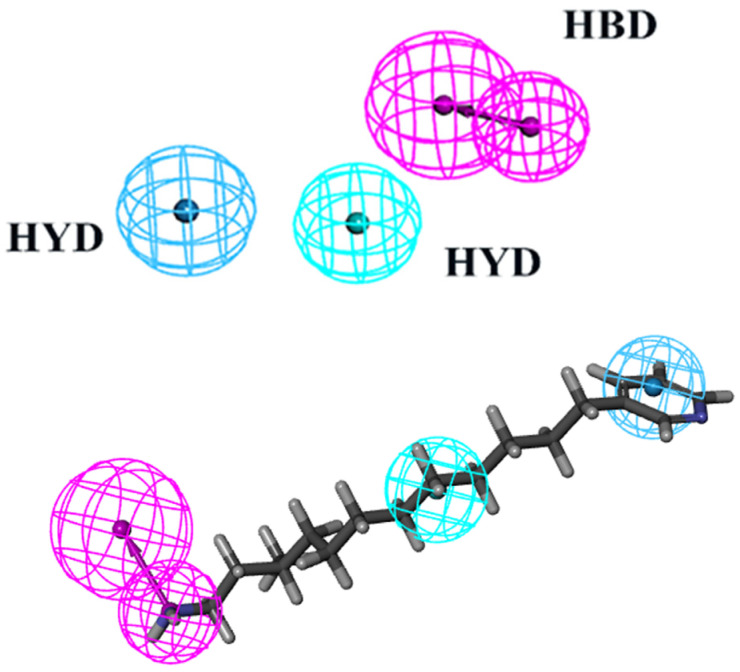
The best HypoGen Pharmacophore model (Hypo1). Green color represents HY; purple color represents HBD; blue color represents HY-aromatic.

The virtual screening process heavily relies on pharmacophore models to illustrate the active conformations of ligand molecules. This is achieved through conformational search and molecular overlay strategies, which help to elucidate the potential interactions between receptor and ligand molecules. Table 1 presents statistics such as cost, correlation coefficient, and RMSD used in the creation of pharmacophores. Ten pharmacophores, each containing HBA, HBD, and HYD features, were produced, with costs ranging from USD 363.483 to USD 374.325, a null cost of USD 408.796, and a fixed cost of USD 301.241. The optimal hypothesis typically has the most significant cost difference, highest correlation coefficient, lowest RMSD, and greatest total cost. According to Table 1, Phar01, the optimal pharmacophore, has the lowest total cost (USD 363.483), highest cost difference (USD 45.313), lowest RMSD (1.216), and best correlation coefficient (0.964). The low RMSD and high correlation coefficient suggest that Phar01 is highly predictive of the experimental activity of compounds in the training set. The characteristics of HBA, HBD, HYD, and other compounds in the training set exhibit an even distribution pattern (Figure 2). The ocean drug library was then used for screening, and 500 molecules with fit values exceeding 4.81 were chosen for further investigation.

### 2.2. ADMET Analysis

**Figure 3:**
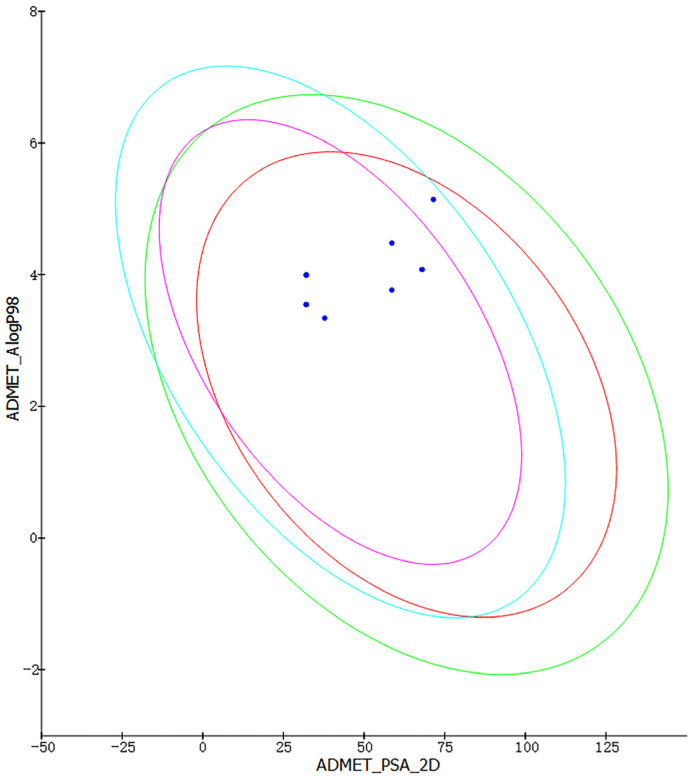
Intestinal absorption model and blood–brain barrier permeability model. (The red ellipses represent 95% confidence intervals for the HIA model, and the green ellipses represent 99% confidence intervals for the HIA mode. Purple ellipses represent 95% confidence intervals for the BBB model, and sky-blue ones represent 99% confidence intervals for the BBB model. Blue dots represent the values of the ADMET_PSA_2 D and the ADMET_AlogP98 for the eight molecules).

We used Discovery Studio to create a predictive procedure. We analyzed the ADMET descriptors of 500 marine molecules that were chosen based on their pharmacophore. After screening, we chose to display the eight molecules with excellent pharmacogenicity. As seen in Figure 3, all eight molecules are within the 99% confidence intervals of both the BBB and HIA models, which shows the accuracy of the model predictions. The ADMET properties of the eight marine molecules with good drug-like properties are shown in Table 2. A BBB level greater than 1 indicates high blood–brain barrier permeability (brain–blood ratio between 1:1 and 1:5). A solubility level of three implies −4.1 < log (Sw) < −2.0, which is indicative of good drug-like properties. An absorption level of 0 suggests good absorption in the human intestine. For hepatotoxicity and CYP2D6 enzyme inhibition, the negative values of the compounds are inversely proportional to the cytochrome enzyme inhibition activity, meaning that the molecules have low hepatotoxicity and cytochrome enzyme inhibition. We selected three molecules without plasma protein-binding capacity, namely compound **1**, compound **2**, and compound **4**, based on plasma protein binding as a reference.

### 2.3. Replace Fragment Protocol

We observed compounds **1**, **2** and **4** from the pharmacophore model and ADMET screening and noticed that the aromatic rings of these molecules had poor interaction with protein PLK1. Thus, we used the Replace Fragment module of the Discovery Studio platform to perform backbone migration, which resulted in 790 compounds. Out of these, 98 small molecules with good drug ability and activity were chosen for further study.

### 2.4. Molecular Docking

**Figure 4:**
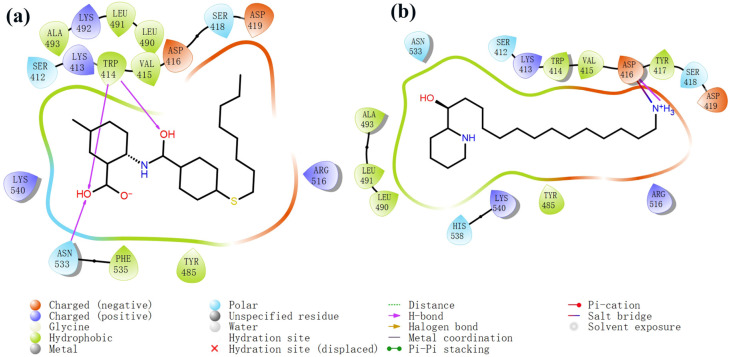
Two-dimensional images of PLHSpTA and marine natural compound 90 interacting with proteins. (**a**) Two-dimensional structure of the protein complex of compound PLHSpTA. (**b**) Two-dimensional structure of marine naural compound **90** and protein complex.

**Figure 5:**
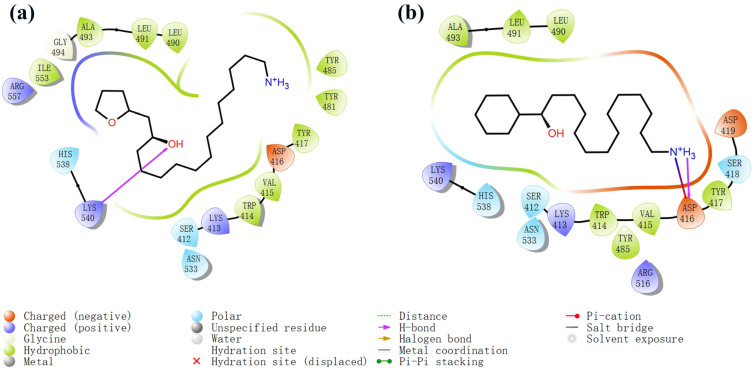
Two-dimensional images of marine natural compound **95** and marine natural compound **97** interacting with proteins. (**a**) Two-dimensional structure of the protein complex of marine natural compound **95**. (**b**) Two-dimensional structure of the marine natural compound **97** protein complex.

**Figure 6:**
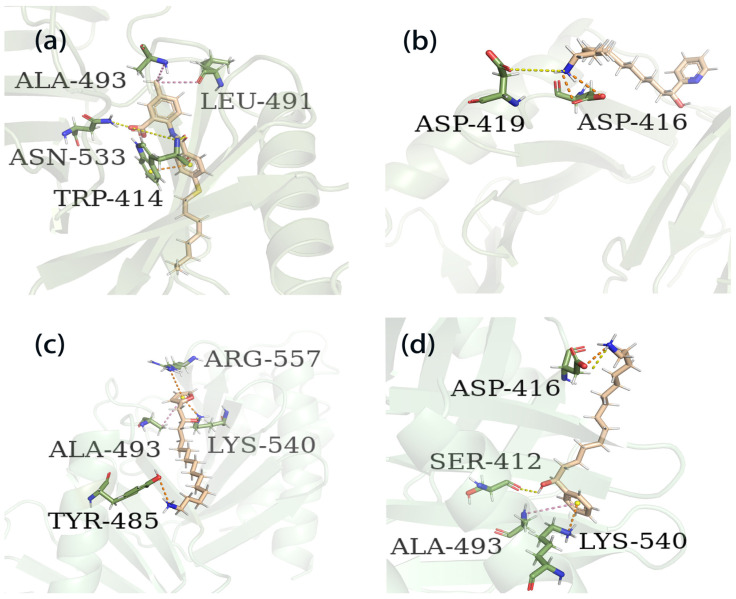
Three-dimensional binding patterns between protein–ligand complexes. (**a**) Three-dimensional structure of the protein complex of compound PLHSpTA. (**b**) Three-dimensional structure of marine natural compound **90** and protein complex. (**c**) Three-dimensional structure of the protein complex of marine natural compound **95**. (**d**) Three-dimensional structure of the marine natural compound **97** protein complex. Hydrogen bond interactions are yellow; electrostatic interaction are orange; hydrophobic interactions are pink.

**Figure 7:**
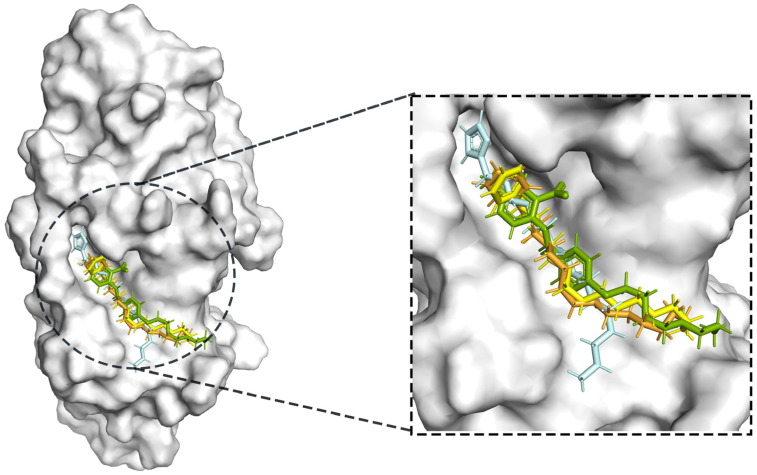
Compound PLHSpTA (the positive control compound) superimposed with the pockets of selected marine natural compounds **90**, **95**, and **97**.

The process of molecular docking involves computer simulation the recognition of molecules in order to identify the optimal binding conformation of proteins and their ligands and to ensure the lowest binding free energy of the complex. Abbapolin [16], a new inhibitor of modified PLHSpTA, was used as the positive compound, and -CDOCKER_ENERGY was used as the scoring criterion. The docking score was higher than or equal to that of the molecule with higher activity than abbapolin, and three molecules with higher scores than the positive compound were identified, namely molecules **90**, **95**, and **97**. The docking scores for these molecules are presented in the Table 3. In molecular docking, scoring functions are important, but it is not reasonable to make judgments based solely on scoring functions. In order to screen for effective PLK1 inhibitors, it is necessary to consider the conformation of the protein–ligand complex and whether there is a clear binding affinity and specificity in the binding pocket in order to reduce the accessibility of substrates and thus inhibit the activity of PLK1. From Figure 4, Figure 5 and Figure 6, we can clearly see the binding modes of these compounds with the PLK1 protein (PDB ID: 3C5L). The analysis revealed ASP416, ASP493, and Lys540 as key residues that contribute to the specific and selective binding to the shallow binding pocket on the PBD. This binding is primarily facilitated through interactions with the di-anionic phosphate group [17,18,19]. The remaining residues (Arg557, Tyr485, and Ala493) were also reported in the paper [20]. As shown in Figure 7, the positive control compound and three other small molecules from marine natural compounds, namely **90**, **95**, and **97**, are simultaneously overlaid on the substrate binding pocket. Structural information for the three selected molecules is shown in Table 4.

### 2.5. Molecular Dynamics

**Figure 8:**
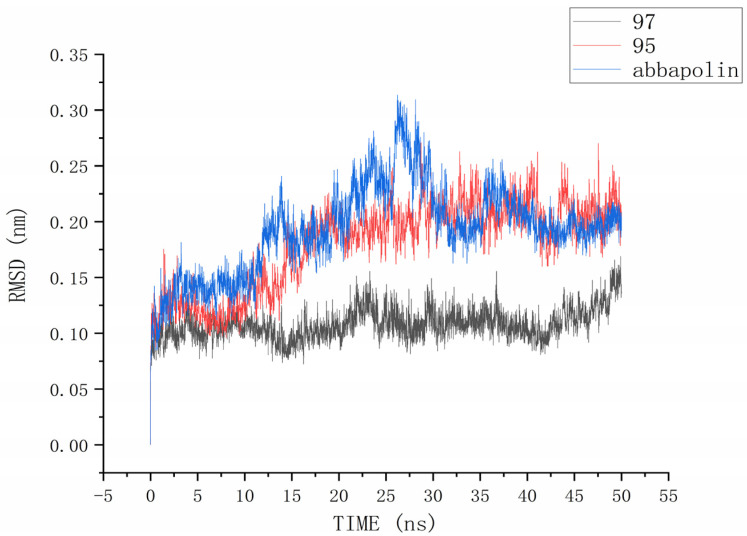
RMSD analysis of the complexes formed by protein PLK1 and three ligands, respectively.

**Figure 9:**
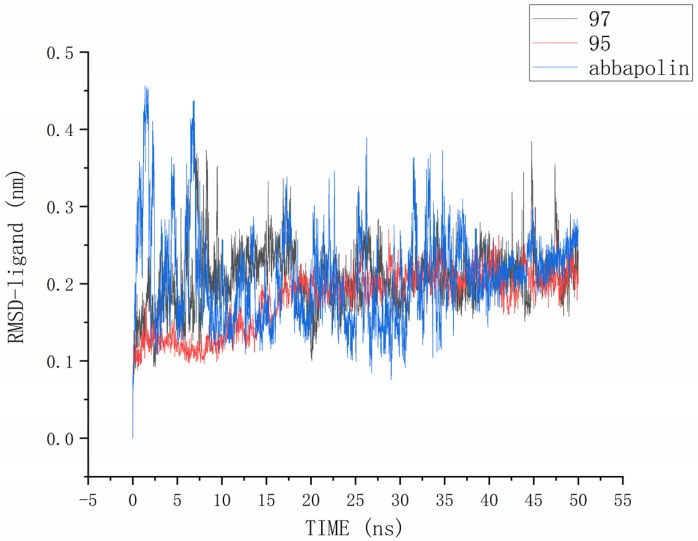
RMSD analysis of three ligands in protein PLK1.

**Figure 10:**
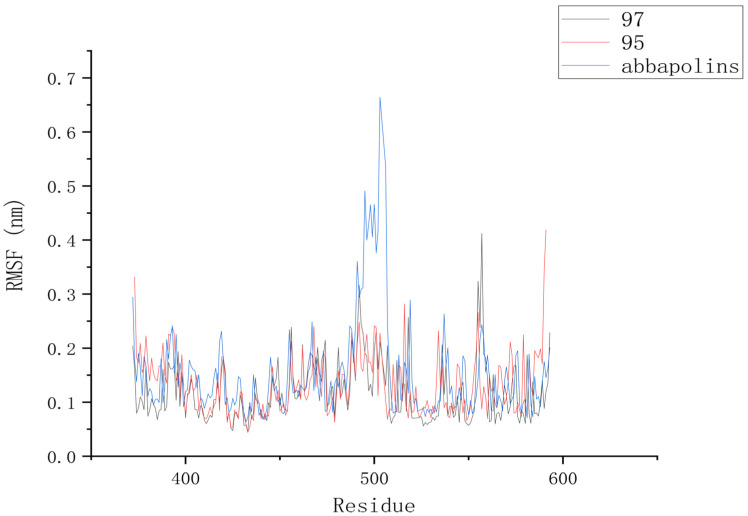
RMSF diagram of PLK1 with compounds.

The root mean square deviation (RMSD) reflects the extent to which atoms deviate from their average positions, indicating the magnitude of motion for each atom. For compound **90**, the significant fluctuations in ligand RMSD raise concerns about the possibility of removing it from the binding pocket. The ligand root mean square deviation (RMSD) for the protein–ligand complex, protein, and positive compound complex is shown in Figure 8. The root means square deviation (RMSD) for **95** stabilizes at 20 ns, with an average RMSD of 0.20522 nm from 20 ns to 50 ns. The root means square deviation (RMSD) for **97** stabilizes at 24 ns, with an average RMSD of 0.11228 nm from 24 ns to 50 ns. The root mean square deviation (RMSD) for abbapolin stabilizes after 35 ns, with an average RMSD of 0.22468 nm from 35 ns to 50 ns. The root mean square deviation (RMSD) for all three ligands stabilizes at equilibrium, with the positively charged compound showing larger fluctuations. Only the RMSD fluctuation for ligand **95** is less than 0.2 nm. Furthermore, compared to the abbapolin–protein complex, the fluctuations in the **95**–protein and **97**–protein complexes are smaller, or the root mean square deviation (RMSD) is smaller. This leads us to further consider that **90** and **97** have better binding stability. The root mean square deviation (RMSD) for the protein backbone is depicted in Figure 9. The average root mean square deviation (RMSD) for the protein backbone in the complexes is 0.19257 nm, 0.18021 nm, and 0.10882 nm, respectively. The root mean square fluctuation (RMSF) of protein residues reflects the displacement of residues in the protein conformation, indicating the freedom of the atoms. As shown in Figure 10, the root mean square fluctuation (RMSF) for all three complexes ranges from 0.0481 nm to 0.4152 nm. Overall, the trend of RMSF changes is consistent, gradually decreasing and stabilizing during the simulation process. However, the root mean square fluctuation (RMSF) value for the positive compound abbapolin is higher than that of compounds **97** and **95,** with a peak occurring at residues 480 to 500. The root means square deviation (RMSD) and root mean square fluctuation (RMSF) data indicate that compounds **97** and **95** demonstrate greater stability in binding to the protein compared to the positive compound.

**Figure 11:**
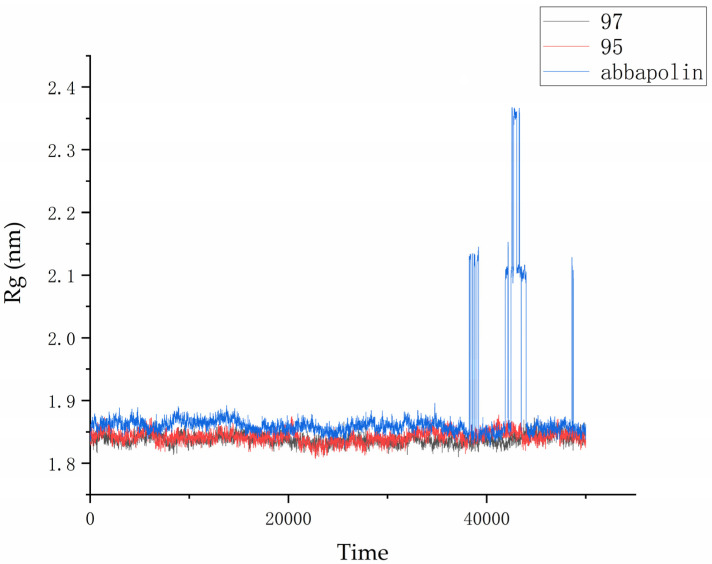
Radius of gyration (Rg) graph for compound **97**, compound **95**, and abbapolin complexes with respect to 50 ns of molecular dynamics.

**Figure 12:**
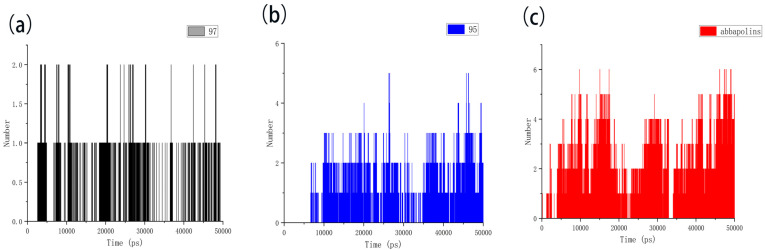
The hydrogen bond of protein with compounds. (**a**) Compound **97** with protein. (**b**) Compound **95** with protein. (**c**) Abbapolin with protein.

The radius of gyration (Rg) is directly related to the compactness of proteins. Therefore, in the presence of positive compounds, i.e., ligand **95** and ligand **97,** the g_gyrate tool in GROMACS was used to monitor the compactness of proteins through the radius of gyration [21]. The Rg results show that the conformation of the PLK1–ligand complex is stable. From Figure 11, it is clear that within the molecular dynamics time of 0 to 37 ns, the Rg value of the PLK1–abbapolin complex fluctuates between 1.83 to 1.88 nm, and there is a larger fluctuation between 37 to 50 ns, while the Rg value of the PLK1–ligand complex is smaller (1.80 to 1.87 nm) and very stable. Hydrogen bonds are the strongest non-covalent interactions and play an important role in the stability of protein–ligand complexes. We analyzed the number of hydrogen bonds in protein–ligand complexes and protein–positive compound complexes over a 50 ns molecular dynamics time span, as shown in Figure 12. The results indicate that positive compounds have a higher number of hydrogen bonds compared to the ligand, but the overall difference is not significant. Ligand **95** has a similar number of hydrogen bonds to the positive compound, while ligand **97** has fewer hydrogen bonds.

### 2.6. Calculation of Binding Free Energy

**Figure 13:**
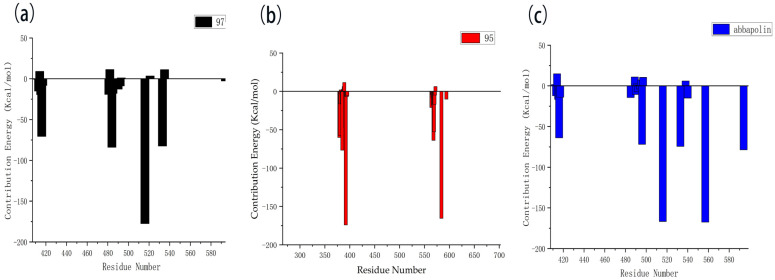
Residue decomposition diagram of binding energy. (**a**) Molecule **97** with protein PLK1. (**b**) Molecule **95** with protein PLK1. (**c**) Abbapolin with protein PLK1.

The results of the free energy analysis are presented in Table 5. The ΔTOTAL for ligands **95** and **97** is −24.57 kcal/mol and −25.97 kcal/mol; both were lower than the ΔTOTAL of abbapolin, although molecule **95** has a binding ability that exceeds abbapolin with great standard deviation. However, it cannot be proven that the binding capacity is higher than abbapolin. The nearly 10 kcal/mol gap shows that the binding free energy of the three is similar. We also decomposed the binding free energy of MM-PBSA into the energy contribution of each protein residue to evaluate the key binding residues, that is, the residues with higher energy contribution to the binding free energy. As shown in Figure 13, the key residues in compound **97** and PLK 1, respectively, are the residues of ARG483, ARG516, ARG557, and ASP416. Interestingly, residue ASP416 forms hydrogen bond interactions during molecular docking, in which ARG557 appears. For PLK1 and compound **95**, its key residues include ARG392, ARG584, and GLU568. The results show that MM-PBSA not only validates the results of molecular docking but also further quantifies the binding energy between the protein PLK 1 and molecules **90** and **95.**

## 3. Discussion

PLK1 is regarded as a promising target for the development of anti-tumor drugs [22]. The sequence features an N-terminal serine/threonine kinase domain and a C-terminal repeat sequence of the polo-box domain (PBD) [23]. Both PLK1 and PBD are autonomous drug targets used in the creation of inhibitors. BI2536, which targets the ATP-binding site (kinase domain), was developed, but its anti-tumor effect was found to be inadequate in clinical trials, leading to its discontinuation. Volasertib has made significant progress and is currently in phase 3 clinical trials. However, most inhibitors that target this binding domain are still in the preclinical stage and have had limited success, likely due to their lack of specificity and the dose-limiting toxicities they cause. On the other hand, drugs that target the PBD-binding domain have the potential to provide greater selectivity at non-toxic doses by focusing on a distinct binding site, thus reducing off-target effects. Allosteric inhibitors can address problems caused by mutations in the conserved ATP binding site by blocking proteins when they are in an inactive state. Targeting protein–protein interactions through PBD has the potential to be more selective. The development of PBD inhibitors could expand the range of cancer treatments available. Several peptide inhibitors of PBD, such as thymoquinone (TQ) and its derivative, poloxin, PLHSpT, and T521, have been studied and have shown promising effects on PBD cell function in vitro. Despite their potential, these compounds often have a short half-life, unpredictable bioavailability, poor stability, and limited membrane permeability, posing challenges for their use in preclinical applications [24,25]. Consequently, there is an immediate need to tackle these major limitations and improve the drug qualities of the inhibitors. To sum up, PBD1 is a possible and appealing target for the formation of extremely selective anti-PLK1 inhibitors for cancer treatment. Gaining a more comprehensive comprehension of the binding mechanism of PBD inhibitors could open up novel opportunities for the advancement of potent PBD1 inhibitors. There is a strong possibility that a novel class of PBD inhibitors will result in considerable advancements in cancer research and precision therapy in the future [26].

Given the expansive marine environment, organisms living in it are considered to be a great resource for bioactive natural products, and the compounds obtained from them are a representation of their rich biodiversity [14]. In recent years, the marine environment has become a focus of new research areas and clinical trials due to the full exploitation of other resources, leading to the development of numerous drugs derived from marine natural products [27,28]. Recent studies have demonstrated that 170 marine natural products and their artificial counterparts possess a powerful anti-cancer action. These marine-based compounds have a unique structural makeup when compared to other naturally occurring substances and can also be used to treat bacterial, viral, and inflammatory conditions. This has led to a growing interest in marine natural products as a potential source of novel medicines. The process of selecting marine compound libraries is a crucial aspect for generating novel research [29].

This research utilized computer-aided drug design techniques to enhance the effectiveness of marine compounds through scaffold hopping. A 3D quantitative structure–activity relationship (3DQSAR) pharmacophore model was constructed and validated using 112 small molecules with specific activity. This pharmacophore model was then used to screen a library of marine compounds, from which 500 molecules were subjected to ADMET prediction. Three of these molecules were identified as having excellent drug-like properties and were chosen for further research. Subsequently, the three molecules underwent scaffold hopping, resulting in the generation of 98 novel compounds with improved drug-like properties and potency. Comparing their docking scores to the positive compound abbapolin, molecules **90**, **95,** and **97** were found to have high activity. Molecular dynamics simulations and MMPBSA were conducted to assess the stability of the compounds when interacting with proteins. The results showed that molecules **95** and **97** had increased stability, suggesting their potential as promising PLK1-PBD inhibitors. Nevertheless, the study has certain limitations. Further research and validation are needed to evaluate the selectivity of the drug molecules. Additionally, further research is required to validate free energy, which will be conducted in the future.

## 4. Materials and Methods

### 4.1. Compound Preparations

First, a thorough search was conducted to identify known PLK1 inhibitors with reported IC50 activity, resulting in the collection of 112 active small molecules from various literature sources. The small molecules collected in SMILES format were subsequently converted to SDF format using StoneMND Collector (StoneWise, Beijing, China, https://stonemind.stonewise.cn/, accessed on 13 July 2023). The small molecules underwent processing using the Prepare Ligand for QSAR tool in Discovery Studio, leading to the generation of 112 small ligand molecules. The Generate Training and Test Data algorithm in Discovery Studio was employed to partition 112 compounds [30,31,32,33,34,35] into a training set and a test set, with a training set percentage of 80. Subsequently, a total of 90 compounds were acquired for the training set, while 22 compounds were obtained for the test set. The training set was utilized for the generation of a pharmacophore model, whereas the test set was employed for the assessment of the predictive capacity of the generated pharmacophore model. Furthermore, this study obtained 52,765 marine drug small-molecule compounds from a natural marine drug database and subsequently performed molecular preparation using the LigPrep tool in the Discovery Studio module.

### 4.2. Pharmacophore Model Generation

The pharmacophore models produced through the 3D-QSAR pharmacophore generation protocol are linked to distinct chemical characteristics that are crucial for molecular bioactivity. The Feature Mapping protocol in Discovery Studio was employed to identify distinct chemical features present on the molecules in the training set. These features, namely hydrogen bond acceptor (HBA), hydrogen bond donor (HBD), hydrophobic (HYD), and ring aromatic (RA), were chosen for the 3D-QSAR pharmacophore generation process. The FAST algorithm was utilized to produce satisfactory conformations for each compound, using an energy threshold of 10 kcal/mol, resulting in a maximum of 255 generated conformations. The uncertainty for the training and test sets was established at 1.5. The IC50 value of individual training set compounds was chosen as the activity attribute during the pharmacophore generation process, while the energy threshold was held constant at 20 kcal/mol. The minimum feature distance was established at 2.97, while the maximum excluded volume was designated as zero. The pharmacophore model was developed using important statistical parameters including total cost value, cost difference, error, root mean square deviation (RMSD), correlation coefficient (r^2^), and pharmacophore features.

### 4.3. Validation of a Pharmacophore Model

To assess the predictive capacity of the pharmacophore model we developed for accurately forecasting the activity of molecules and identifying active compounds from a database, we employed cost analysis and test set analysis to validate the model. The HypoGen module in Catalyst provides information on three categories of costs: fixed cost, zero cost, and total cost. The term “fixed cost”, also referred to as ideal cost, denotes the most basic model that can accurately accommodate all the data. “Zero cost”, also referred to as unrelated cost, denotes the maximum cost of a pharmacophore lacking features and is assessed by averaging the molecular activity data of the training set. Typically, a discrepancy of 40–60 bits between the overall cost and zero cost suggests a 75–90% probability of accurately reflecting the correlation in the data. A test set comprising 34 compounds, which are structurally distinct from the training set and exhibit a broad spectrum of activity values, was employed.

### 4.4. Database Searching

The virtual screening of chemical databases can serve to discover new and suitable virtual lead compounds with potential for further development. The advantage of database retrieval methods is that the compounds retrieved can be easily used for biological testing as compared to de novo design methods. Pharmacophore models can be used for structure searching, searching for the structure of composite pharmacophore models in 3D databases, and predicting the activity of new compounds. Pharmacophore models are commonly used in 3D databases, where a molecule must meet all the features of the pharmacophore model to be retained as a hit. In DS, there are two search methods: Fast/Flexible and Best/Flexible, with Best/Flexible having higher accuracy and yielding better results. In this experiment, we chose the Best/Flexible search option to retrieve our database.

### 4.5. ADMET

The pharmacokinetic method of ADMET (drug absorption, distribution, metabolism, excretion, and toxicity) is important in drug design and drug screening. The prediction of the ADMET properties of drugs can effectively guide the structural optimization and transformation, improve the success rate of drug research and development, and reduce the cost of drug research and development. We analyzed the ADMET descriptors of marine molecules selected by pharmacophore. This research was carried out through the Calculate Molecular Properties function of the Discovery Studio platform. The blood–brain barrier permeability (BBB), water solubility, intestinal absorbance, hepatotoxicity, plasma protein binding, and CYP2D6 enzyme inhibition descriptors of the drug were predicted.

### 4.6. Scaffold Hopping Replace Fragment Protocol

Scaffold hopping is the process of replacing the core skeleton of a ligand with a new moiety with a similar function to improve the properties of a compound or to find completely new compounds with similar functions. The Replace Fragment protocol identifies isosteric fragments from the default fragment libraries, and the original fragment is automatically replaced to create novel ligands. Fragments are judged isosterically if they are similar to the original fragment, where similarity is defined by the user through the selected molecular properties and fingerprints. By default, the properties of number of rings, number of aromatic rings, and molecular surface area are used to calculate the similarity as Euclidean distance. These properties have been shown to give the best balance between chemotype diversity and isofunctional similarity with the original lead compound [36]. In this study, the Replace Fragment module of the Discovery studio platform was used to generate potential replacement fragments based on protein active sites, namely small-molecule structures.

### 4.7. Molecular Docking

Molecular docking is a technique that can be utilized to investigate the most favorable binding conformation between compounds and their respective targets. Hence, to enhance the screening of compounds with favorable target inhibition activity, we employed the PLK1 (PDB ID: 3C5L) protein structure as the target for molecular docking by using the CDOCKER module in Discovery Studio. We downloaded the PLK1 protein (PDB ID: 3C5L) from the Protein Data Bank (PDB) website (https://www.rcsb.org/, accessed on 27 August 2023). We utilized the Protein Preparation Wizard tool in Maestro 11.8 to prepare the protein molecule, which involved assigning bond orders, hydrogenating, and eliminating water molecules beyond 5 Å during the initial processing. To further refine the protein molecule, we optimized the protonation states of residues at pH 7.0 using PROKA. Furthermore, we collected the heavy atoms of the protein molecule with an RMSD of 0.3 Å and then minimized the protein molecule using the OPLS_2005 force field. The binding site of PLK1 (PDB ID: 3C5L) is determined by the original ligand, with the center coordinates of the docking site being 5.79976, 33.8292 and 58.4005, 8.94014. The docking radius was established at 17, with the original ligand serving as the focal point, and a sphere with a docking radius of 10 was designated as the active site. The docking priority was configured to high quality, the spatial hot-spot number was set to 100, and the ligand conformation generation method was specified as BEST for the operation. To differentiate and ascertain the molecules with superior target binding activity, we opted for the novel inhibitor abbapolin [16], which exhibits inhibitory activity, for our molecular docking studies. Compounds with docking scores exceeding a certain threshold were deemed to warrant further research. Prediction of the structural information of the three selected molecules was performed using ChemDraw 14.0.

### 4.8. Molecular Dynamics

The text describes the evaluation of the atomic stability of different ligands on the protein PLK1 using molecular dynamics with the AMBER99SB-ILDN force field in GROMACS [37,38]. The ligands **90**, **95**, and **97** and the positive compounds were analyzed in a 50 ns molecular dynamics simulation with the protein receptor system. The protein was processed using the AMBER99SB-ILDN force field to generate topology and coordinate files. The Bio2byte web server (https://www.bio2byte.be/, accessed on 17 October 2023) was used to generate topology file for molecules A cubic box with a radius of 1.5 nm and the SPC216 water model were used to define periodic boundary conditions (PBC). Additionally, the genion tool was used to neutralize the system by adding counter ions (Na^+^ ions and Cl^−^ ions). The final system contains 200,059 water molecules and two Cl^−^ ions. All systems underwent energy minimization using the steepest descent algorithm for 50,000 steps. The systems were equilibrated at 300 K in two steps: constant particle number, volume, and temperature (NVT) and constant particle number, pressure, and temperature (NPT) for 1 ns. During the equilibration process, temperature and pressure were regulated by the Berendsen thermostat and Parrinello–Rahman barostat, respectively. Long-range electrostatic interactions and covalent bonds were maintained using the particle mesh Ewald (PME) and the linear constraint solver (LINCS) methods. The final molecular dynamics simulation ran for 50 ns, with file updates at 10 ps intervals [39,40,41].

### 4.9. MM-PBSA

The gmx_MMPBSA 1.5.2 package was used to calculate the free energy using the MM/PBSA (molecular mechanics/Poisson–Boltzmann surface area) method [42]. The source of input data was the trajectory (last 80% of frames) generated by GROMACS in the process of molecular dynamics simulation of target associates. The dielectric interface was implemented using the level-setting function. At the same time, nonpolar solvation free energy was modeled with SASA (solvent accessible surface area). The external dielectric constant was equal to 80, and the internal dielectric constant was 2. The contribution of the entropy component was calculated by the IE (interaction entropy) method. 

## 5. Conclusions

PLK1 is considered a key target for cancer treatment. In this study, a 3D quantitative structure–activity relationship (3DQSAR) pharmacophore model was created and validated using 112 small molecules with specific activity. The model was then used to screen a library of marine compounds, resulting in the identification of 500 molecules for ADMET prediction. Three molecules with favorable drug-like properties were chosen for further investigation. These molecules underwent scaffold hopping, resulting in 98 new compounds with improved drug-like properties and increased potency. Molecular docking was carried out, and the compound abbapolin was identified as a promising candidate, confirming the high activity of molecules **90**, **95**, and **97**. Molecular dynamics simulations were then conducted to assess the stability of the compounds with proteins, and the binding energies of the ligand–protein complexes were calculated. Analysis revealed that molecule **95** and **97** show potential as novel PLK1 inhibitors, opening up new possibilities for the targeted treatment of associated cancers.

## Figures and Tables

**Table 1:** Statistical results of the top 10 pharmacophore hypotheses generated by the HypoGen algorithm.

Pharmacophore	Features	Total Cost (USD)	Cost Difference (USD)	Correlation	RMSD	Max.Fit
Phar01	HHD	363.483	45.313	0.964	1.216	7.222
Phar02	HHD	370.353	38.443	0.448	1.274	4.30
Phar03	HHD	370.457	38.339	0.364	1.290	6.375
Phar04	HAA	372.205	36.591	0.828	1.290	4.239
Phar05	HDD	374.201	34.595	0.383	1.326	5.840
Phar06	HAD	374.325	34.471	0.165	1.323	5.146
Phar07	HAD	374.473	34.323	0.137	1.325	5.234
Phar08	ADD	375.973	32.823	0.457	1.340	6.509
Phar09	HAA	376.062	32.734	0.229	1.319	3.930
Phar10	HAAD	376.754	32.042	0.400	1.309	4.436

**Table 2:** ADME properties of the top eight compounds in similarity scoring.

Compound	BBB Level	Solubility	Absorption Level	Hepatotoxic	CYP2D6 Inhibit	Plasma Protein-Binding Prediction
			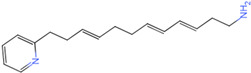			
**1**	1	3	0	−7.00099	−3.01411	False
			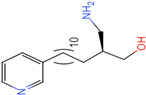			
**2**	1	3	0	−6.3594	−4.63198	False
			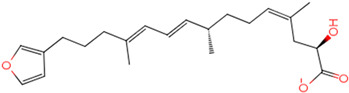			
**3**	1	3	0	−7.1642	−5.51768	True
			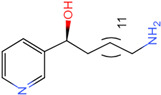			
**4**	1	3	0	−7.42021	−1.2257	False
			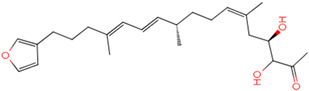			
**5**	1	3	0	−7.25533	−5.44008	True
			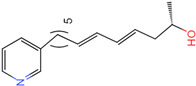			
**6**	1	3	0	−6.13045	−3.11628	True
			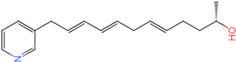			
**7**	1	3	0	−7.34797	−2.16912	True
			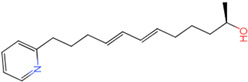			
**8**	1	3	0	−6.43977	−1.8575	True

**Table 3:** Docking scores for the three selected molecules and the positive compounds.

Molecules	-CDOCKER_ENERGY
Molecule **90** [(14S)-14-hydroxy-14-(pyridin-2-yl)tetradecyl]ammonium	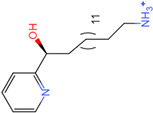 44.0031
Molecule **95** [(14S)-15-(2-furyl)-14-hydroxypentadecyl]ammonium	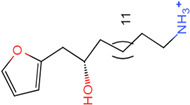 43.9085
Molecule **97** [(14S)-14-hydroxy-14-phenyltetradecyl]ammonium	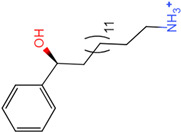 39.3765
Abbapolin	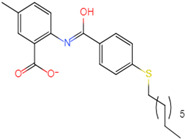 41.8117

**Table 4:** Structural information of the three selected molecules.

Molecule	Chemical Formula	Exact Mass	Mol Weight	*m*/*z*	Elemental Analysis	Log P	Henry’s Law	tPSA	Clog P	CMR	LOG S	pKa
**90**	C19H35N2O+	307.274	307.501	307.274 (100.0%), 308.277 (20.5%), 309.281 (2.0%)	C,74.21;H,11.47:N,9.11:; O,5.20	4.71	0.69	60.23	4.417	9.3151	−4.652	10.324.13.885
**95**	C19H36NO2+	310.274	310.501	310.274 (100.0%), 311.277 (20.5%), 312.280 (2.0%)	C,73.50;H,11.69:N,4.51: 0,10.31	_	−1.32	57.1	5.319	9.2039	−4.45	10.324.14.308
**97**	C20H36NO+	306.279	306.513	306.279 (100.0%), 307.282 (21.6%), 308.285 (2.2%)	C,78.37:H,11.84:N,4.57: 0,5.22	5.59	−0.67	47.87	5.914	9.5262	−5.429	10.324.14.257

**Table 5:** Molecular mechanic/Poisson–Boltzmann surface area (MMPBSA) complexes calculated from 1000 frames (40–50 ns) of molecular dynamics. All the binding free energies, i.e., van der Waals energy, electrostatic energy, molecular mechanics term (energy in gas phase), energy of nucleus of solvation, and total binding free energy, are shown in kJ/mol.

Molecules	ΔVDWAALS	ΔEEL	ΔGSOLV	ΔGGAS	ΔTOTAL
Molecule 95	−18.41 ± 0.93	−30.2 ± 12.86	24.04 ± 12.90	−48.6 ± 12.90	−24.57 ± 18.24
Molecule 97	−28.53 ± 0.47	52.38 ± 0.31	23.85 ± 0.57	−49.82 ± 0.95	−25.97 ± 1.11
Abbapolin	−47.15 ± 0.94	−175.79 ± 4.90	171.96 ± 3.48	−210.94 ± 4.98	−38.98 ± 6.08

## Data Availability

The original data presented in the study are included in the article; further inquiries can be directed to the corresponding author.

## References

[1] Chen C., Zhang M., Pan X., Yuan X., Zhou L., Yan L., Zeng L.-H., Xu J., Yang B., Zhang L. (2021). Bub1 and CENP-U Redundantly Recruit Plk1 to Stabilize Kinetochore-Microtubule Attachments and Ensure Accurate Chromosome Segregation. Cell Rep..

[2] Peng Y., Liu Y., Zheng R., Ye Y., Fu Y., Yin L., Gao Y., Fu Y., Qi X., Deng T. (2023). PLK1 Maintains DNA Methylation and Cell Viability by Regulating Phosphorylation-Dependent UHRF1 Protein. Cell Death Discov..

[3] Luo P., Yan H., Du J., Chen X., Shao J., Zhang Y., Xu Z., Jin Y., Lin N., Yang B. (2021). PLK1 (Polo like Kinase 1)-Dependent Autophagy Facilitates Gefitinib-Induced Hepatotoxicity by Degrading COX6A1 (Cytochrome c Oxidase Subunit 6A1). Autophagy.

[4] Zhang J., Zhang L., Wang J., Ouyang L., Wang Y. (2022). Polo-like Kinase 1 Inhibitors in Human Cancer Therapy: Development and Therapeutic Potential. J. Med. Chem..

[5] Karaman M.W., Herrgard S., Treiber D.K., Gallant P., Atteridge C.E., Campbell B.T., Chan K.W., Ciceri P., Davis M.I., Edeen P.T. (2008). A Quantitative Analysis of Kinase Inhibitor Selectivity. Nat. Biotechnol..

[6] Vaughan C.A., Singh S., Subler M.A., Windle J.J., Inoue K., Fry E.A., Pillappa R., Grossman S.R., Windle B., Andrew Yeudall W. (2021). The Oncogenicity of Tumor-Derived Mutant P53 Is Enhanced by the Recruitment of PLK3. Nat. Commun..

[7] Gao Y., Kabotyanski E.B., Shepherd J.H., Villegas E., Acosta D., Hamor C., Sun T., Montmeyor-Garcia C., He X., Dobrolecki L.E. (2021). Tumor Suppressor PLK2 May Serve as a Biomarker in Triple-Negative Breast Cancer for Improved Response to PLK1 Therapeutics. Cancer Res. Commun..

[8] Sabe V.T., Ntombela T., Jhamba L.A., Maguire G.E.M., Govender T., Naicker T., Kruger H.G. (2021). Current Trends in Computer Aided Drug Design and a Highlight of Drugs Discovered via Computational Techniques: A Review. Eur. J. Med. Chem..

[9] Neves B.J., Braga R.C., Melo-Filho C.C., Moreira-Filho J.T., Muratov E.N., Andrade C.H. (2018). QSAR-Based Virtual Screening: Advances and Applications in Drug Discovery. Front. Pharmacol..

[10] Zeng S., Huang W., Zheng X., Liyan C., Zhang Z., Wang J., Shen Z. (2021). Proteolysis Targeting Chimera (PROTAC) in Drug Discovery Paradigm: Recent Progress and Future Challenges. Eur. J. Med. Chem..

[11] Barreca M., Spanò V., Montalbano A., Cueto M., Díaz Marrero A.R., Deniz I., Erdoğan A., Lukić Bilela L., Moulin C., Taffin-de-Givenchy E. (2020). Marine Anticancer Agents: An Overview with a Particular Focus on Their Chemical Classes. Mar. Drugs.

[12] Jimenez P.C., Wilke D.V., Branco P.C., Bauermeister A., Rezende-Teixeira P., Gaudêncio S.P., Costa-Lotufo L.V. (2020). Enriching Cancer Pharmacology with Drugs of Marine Origin. Br. J. Pharmacol..

[13] Alves C., Silva J., Pinteus S., Gaspar H., Alpoim M.C., Botana L.M., Pedrosa R. (2018). From Marine Origin to Therapeutics: The Antitumor Potential of Marine Algae-Derived Compounds. Front. Pharmacol..

[14] Luo L., Wang Q., Liao Y. (2022). The Inhibitors of CDK4/6 from a Library of Marine Compound Database: A Pharmacophore, ADMET, Molecular Docking and Molecular Dynamics Study. Mar. Drugs.

[15] Lyu C., Chen T., Qiang B., Liu N., Wang H., Zhang L., Liu Z. (2021). CMNPD: A Comprehensive Marine Natural Products Database towards Facilitating Drug Discovery from the Ocean. Nucleic Acids Res..

[16] Chapagai D., Merhej G., McInnes C., Wyatt M.D. (2023). Structural Basis for Variations in Polo-like Kinase 1 Conformation and Intracellular Stability Induced by ATP-Competitive and Novel Noncompetitive Abbapolin Inhibitors. ACS Chem. Biol..

[17] Elia A.E.H., Rellos P., Haire L.F., Chao J.W., Ivins F.J., Hoepker K., Mohammad D., Cantley L.C., Smerdon S.J., Yaffe M.B. (2003). The Molecular Basis for Phosphodependent Substrate Targeting and Regulation of Plks by the Polo-Box Domain. Cell.

[18] Lowery D.M., Lim D., Yaffe M.B. (2005). Structure and Function of Polo-like Kinases. Oncogene.

[19] Yun S.-M., Moulaei T., Lim D., Bang J.K., Park J.-E., Shenoy S.R., Liu F., Kang Y.H., Liao C., Soung N.-K. (2009). Structural and Functional Analyses of Minimal Phosphopeptides Targeting the Polo-Box Domain of Polo-like Kinase 1. Nat. Struct. Mol. Biol..

[20] Stafford J.M., Wyatt M.D., McInnes C. (2023). Inhibitors of the PLK1 Polo-Box Domain: Drug Design Strategies and Therapeutic Opportunities in Cancer. Expert Opin. Drug Discov..

[21] Yekeen A.A., Durojaye O.A., Idris M.O., Muritala H.F., Arise R.O. (2023). CHAPERONg: A Tool for Automated GROMACS-Based Molecular Dynamics Simulations and Trajectory Analyses. Comput. Struct. Biotechnol. J..

[22] AlAjmi M.F., Rehman M.T., Hussain A., Rather G.M. (2018). Pharmacoinformatics Approach for the Identification of Polo-like Kinase-1 Inhibitors from Natural Sources as Anti-Cancer Agents. Int. J. Biol. Macromol..

[23] Zitouni S., Nabais C., Jana S.C., Guerrero A., Bettencourt-Dias M. (2014). Polo-like Kinases: Structural Variations Lead to Multiple Functions. Nat. Rev. Mol. Cell Biol..

[24] Erak M., Bellmann-Sickert K., Els-Heindl S., Beck-Sickinger A.G. (2018). Peptide Chemistry Toolbox—Transforming Natural Peptides into Peptide Therapeutics. Bioorg. Med. Chem..

[25] Murugan R.N., Park J.-E., Kim E.-H., Shin S.Y., Cheong C., Lee K.S., Bang J.K. (2011). Plk1-Targeted Small Molecule Inhibitors: Molecular Basis for Their Potency and Specificity. Mol. Cells.

[26] Yun T., Qin T., Liu Y., Lai L. (2016). Identification of Acylthiourea Derivatives as Potent Plk1 PBD Inhibitors. Eur. J. Med. Chem..

[27] Matulja D., Wittine K., Malatesti N., Laclef S., Turks M., Markovic M.K., Ambrožić G., Marković D. (2020). Marine Natural Products with High Anticancer Activities. Curr. Med. Chem..

[28] Song C., Yang J., Zhang M., Ding G., Jia C., Qin J., Guo L. (2021). Marine Natural Products: The Important Resource of Biological Insecticide. Chem. Biodivers..

[29] Luo L., Zhong A., Wang Q., Zheng T. (2021). Structure-Based Pharmacophore Modeling, Virtual Screening, Molecular Docking, ADMET, and Molecular Dynamics (MD) Simulation of Potential Inhibitors of PD-L1 from the Library of Marine Natural Products. Mar. Drugs.

[30] Craig S.N., Baxter M., Chapagai D., Stafford J.M., Nurmemmedov E., Altomare D., Wyatt M.D., McInnes C. (2022). Structure-Activity and Mechanistic Studies of Non-Peptidic Inhibitors of the PLK1 Polo Box Domain Identified through REPLACE. Eur. J. Med. Chem..

[31] Abdelfatah S., Berg A., Huang Q., Yang L.J., Hamdoun S., Klinger A., Greten H.J., Fleischer E., Berg T., Wong V.K.W. (2019). MCC1019, a Selective Inhibitor of the Polo-Box Domain of Polo-like Kinase 1 as Novel, Potent Anticancer Candidate. Acta Pharm. Sin. B.

[32] Zhao X.Z., Tsuji K., Hymel D., Burke T.R. (2019). Development of Highly Selective 1,2,3-Triazole-Containing Peptidic Polo-like Kinase 1 Polo-Box Domain-Binding Inhibitors. Molecules.

[33] Zhao X.Z., Hymel D., Burke T.R. (2017). Enhancing Polo-like Kinase 1 Selectivity of Polo-Box Domain-Binding Peptides. Bioorg. Med. Chem..

[34] Hymel D., Grant R.A., Tsuji K., Yaffe M.B., Burke T.R. (2018). Histidine N(τ)-Cyclized Macrocycles as a New Genre of Polo-like Kinase 1 Polo-Box Domain-Binding Inhibitors. Bioorg. Med. Chem. Lett..

[35] Hymel D., Burke T.R. (2017). Phosphatase-Stable Phosphoamino Acid Mimetics That Enhance Binding Affinities with the Polo-Box Domain of Polo-like Kinase 1. ChemMedChem.

[36] Raafat A., Mowafy S., Abouseri S.M., Fouad M.A., Farag N.A. (2022). Lead Generation of Cysteine Based Mesenchymal Epithelial Transition (c-Met) Kinase Inhibitors: Using Structure-Based Scaffold Hopping, 3D-QSAR Pharmacophore Modeling, Virtual Screening, Molecular Docking, and Molecular Dynamics Simulation. Comput. Biol. Med..

[37] Sousa da Silva A.W., Vranken W.F. (2012). ACPYPE—AnteChamber PYthon Parser interfacE. BMC Res. Notes.

[38] Mark P., Nilsson L. (2001). Structure and Dynamics of the TIP3P, SPC, and SPC/E Water Models at 298 K. J. Phys. Chem. A.

[39] Hosseini F.S., Amanlou M. (2020). Anti-HCV and Anti-Malaria Agent, Potential Candidates to Repurpose for Coronavirus Infection: Virtual Screening, Molecular Docking, and Molecular Dynamics Simulation Study. Life Sci..

[40] Van Der Spoel D., Lindahl E., Hess B., Groenhof G., Mark A.E., Berendsen H.J.C. (2005). GROMACS: Fast, Flexible, and Free. J. Comput. Chem..

[41] Nava M. (2018). Implementing Dimer Metadynamics Using Gromacs. J. Comput. Chem..

[42] Wang E., Sun H., Wang J., Wang Z., Liu H., Zhang J.Z.H., Hou T. (2019). End-Point Binding Free Energy Calculation with MM/PBSA and MM/GBSA: Strategies and Applications in Drug Design. Chem. Rev..

